# Does Prolonged Preservation of Blastocysts Affect the Implantation and Live Birth Rate? A Danish Nationwide Register-Based Study

**DOI:** 10.3390/jcm15031072

**Published:** 2026-01-29

**Authors:** Tilde Veng Eskildsen, Michael Due Larsen, Jens Fedder, Line Riis Jølving

**Affiliations:** 1 Fertility Clinic, Odense University Hospital, 5000 Odense, Denmark; jenfed@rm.dk; 2 Clinical Research Unit of Gynaecology and Obstetrics, Institute of Clinical Research, University of Southern Denmark, 5000 Odense, Denmark; 3 Center for Clinical Epidemiology, Odense University Hospital, 5000 Odense, Denmark; michael.d.larsen@ntnu.no (M.D.L.); line.joelving@rsyd.dk (L.R.J.); 4 Department of Clinical and Molecular Medicine, Faculty of Medicine and Health Sciences, Norwegian University of Science and Technology, 7491 Trondheim, Norway; 5 Centre of Andrology & Fertility Clinic, Horsens Hospital & Aarhus University, 8700 Horsens, Denmark; 6 Research Unit of Clinical Epidemiology, University of Southern Denmark, 5000 Odense, Denmark

**Keywords:** frozen embryos, vitrification, storage time, live birth, LGA

## Abstract

**Background/Objectives:** Cryopreservation technology used in assisted reproductive technology (ART) has significantly improved live birth rates by enabling multiple embryo transfers with frozen embryos from a single ovarian stimulation cycle. However, there is conflicting data on the effect of prolonged cryopreservation of human blastocysts. **Methods:** This Danish nationwide cohort study includes all frozen embryo transfers (FETs) from 1 January 2012 to 31 March 2019. Biochemical pregnancy, clinical pregnancy, and live births were analyzed based on blastocyst storage time. Blastocyst storage time was stratified into five groups, ≤3 month, 4–6 months, 7–12 months, 13–24 months, and ≥25 months, with the shortest (≤3 months) as the reference. We also examined the risk of preterm birth, small and large for gestational age (SGA and LGA), and congenital malformations among live-born children. Multivariable analysis was used to estimate the odd ratios of the reproductive outcomes, accounting for potential confounders. **Results:** We identified 7042 women with 12,599 FETs. Characteristics of women at embryo transfer did not vary significantly by storage time, except for polycystic ovarian syndrome (PCOS), which increased from 2.6% in the reference group to 6.7% in the ≥25-month group. The clinical pregnancy rate was 35.7%. Blastocyst storage time did not significantly affect biochemical pregnancy rates, with adjusted odds ratios (aORs) of 0.94 (95% CI: 0.80–1.11) to 0.96 (95% CI: 0.82–1.12) for the 13–24-month and ≥25-month groups, respectively. Clinical pregnancy rates also did not decrease with storage time (aOR 0.96, 95% CI: 0.82–1.13) for ≥25 months. The live birth rate was 28.6%, with no significant decrease during storage (aOR 0.89, 95% CI: 0.75–1.06). However, the risk of LGA was slightly, but non-significantly, increased (aOR: 1.42, 95% CI: 0.84–2.42) in the ≥25-month group, whereas the aOR of SGA and congenital malformations was not increased. **Conclusions:** Our data indicates that storing blastocysts for a period of 25 months does not significantly affect pregnancy chances following assisted reproductive technology treatment.

## 1. Introduction

Since the first assisted reproductive technology (ART) treatment following the successful transfer of a frozen embryo in 1983, the cryopreservation of embryos in vitro has increasingly been used [1,2]. Cryopreservation technology used in ART treatment has significantly improved the live birth rate, allowing multiple embryo transfers with frozen embryos from a single ovarian stimulation cycle. Additionally, the elective freeze of all embryos (freeze-all technique) is becoming more frequent as pregnancy rates after frozen embryo transfers (FETs) are approaching or exceeding those of fresh transfer cycles [3]. Furthermore, the freeze-all strategy offers the advantage of allowing the application of gonadotropin-releasing hormone agonist to finalize oocyte maturation and thus minimize the risk of early and late ovarian hyperstimulation syndrome [3,4]. In Denmark, the number of FET cycles has doubled over 5 years, from 4190 FET cycles in 2015 to 8070 FET cycles in 2020, and is expected to increase further. In other parts of Europe, the proportion of cryopreserved embryo transfers is even higher [5].

Two cryopreservation methods have been used routinely: slow-freeze and vitrification. Slow-freeze allows freezing to occur at a sufficiently slow rate to permit adequate cellular dehydration while minimizing intracellular ice formation. The more recent, rapid vitrification method allows the solidification of the cell(s) and the extracellular milieu into a glass-like state without the formation of ice [6]. Data from available RCTs discussed in a meta-analysis from Rienzi et al. 2017 demonstrate that vitrification/warming is superior to slow-freeze/thawing for oocytes, cleavage-stage embryos, and blastocysts [6]; thus, most clinics have transitioned to the use of vitrification for cryopreservation.

Several studies, with FET cycles divided into different timeline groups, with the shortest storage being <3 months and the longest storage being >8 years, state no differences in pregnancy outcomes nor miscarriage from day 5/6 for good-grade blastocysts after cryopreservation [7,8,9,10,11,12,13]. One study pointed out that postponement of transfer increased the risk of large for gestational age (LGA) birth [9]. Thus, cryopreservation of human embryos in liquid nitrogen (LN_2_) is expected to be stable for years [6,14,15,16,17]. Controversially, equally many studies, with FET cycles divided into similar timeline groups, state opposite results and suggest a negative correlation between storage time and live birth rates after cryopreservation >2 years [18,19,20,21,22,23]. According to these studies, patients who adopt a freeze-all strategy should be advised that early FET might achieve better clinical outcomes.

Meanwhile, with the increase in cryopreserved embryos and the trend of delayed childbearing in many countries, a growing number of cryopreserved embryos will be transferred in the future, so determining the effect of long cryo-storage duration on human embryos is of great importance. Moreover, the maximum length of embryo cryopreservation that does not affect the embryo or the neonatal clinical outcome is unknown, and there might be a difference in the consequences of storage time between the two methods for cryopreservation and between cleavage-stage embryos and blastocysts.

This cohort study investigates the impact of the storage duration of vitrified warmed embryos on reproductive outcomes, i.e., biochemical pregnancy, clinical pregnancy, live birth, and preterm birth, small for gestational age (SGA), and LGA, as well as congenital malformations, in Danish women who underwent FET cycles following freeze-all storage or following a fresh embryo transfer.

## 2. Materials and Methods

### 2.1. Data Collection

This nationwide cohort study utilized data from the Danish health registries, encompassing all FETs performed at public and private clinics in Denmark from 1 January 2012 to 31 March 2019, with follow-up on childbirths until the end of 2020. Denmark’s population of approximately 5.9 million inhabitants is uniquely identifiable through a civil registration number assigned at birth or immigration, facilitating accurate record linkage across health registers [24]. 

We obtained data from (i) The Danish ART Register on information on ART procedures, infertility causes, and pregnancy outcomes [25,26,27] (ii) The Danish National Patient Register (DNPR) on comorbidities [24] and (iii) The Danish Medical Birth Register (DMBR) on outcomes of the ART treatment [28,29], including live births and neonatal outcomes, specifically, preterm birth (defined as birth < 37 weeks of gestation) and small and large for gestational age. SGA was defined as weight < 10th percentile for babies of the same gestational age, and LGA was defined as birth weight > 90th percentile of the same gestational age and congenital malformation (minor or major but with the exclusion of congenital malformations with testicular retention; please refer to Appendix A). Finally, we collected (iv) data on death and migration from The Civil Registration System [24].

Data in the study cohort is a complete dataset, with full follow-up. The adjustment model excludes embryos with missing data of crude character. The only missing data allowed in the study cohort are on alcohol, smoking, and BMI.

### 2.2. ART Procedures

Only good-quality blastocysts according to the Gardner score (Gardner score at minimum 2BB and generally without C-scores in either trofectoderm or inner cell mass) are included in this study; thus, on the fifth or sixth day, the embryos are graded based on morphology according to the Gardner classification system [30,31]. The blastocysts suitable for transfer and vitrification, i.e., according to Gardner score and with the greatest expected implantation potential, are transferred to the patient’s uterus. Surplus embryos with expected implantation potential are cryopreserved, meaning they are frozen and stored until needed for later transfer. In the event of ovarian hyperstimulation syndrome (OHSS), all suitable blastocysts are cryopreserved for later use, thus postponing pregnancy while the woman recovers from OHSS.

As mentioned, two cryopreservation methods were used routinely during the study period: slow-freeze and vitrification. This study includes only vitrified-warmed blastocysts. The vitrification method utilizes high concentrations of cryoprotectant but for a very rapid exposure time, thus circumventing the risk of intracellular ice formation. After cryopreservation, the embryos are stored at a constant temperature of −196 °C in either liquid or gas phase in liquid nitrogen tanks. For the warming process of cryopreserved embryos, the embryos are unloaded from the carriers according to local protocols. For endometrial preparation, a natural cycle was used for patients with regular menstrual cycles, or a stimulation cycle was used for patients with irregular menstrual cycles [32]. In some clinics, progesterone supplementation was provided until 8 weeks of gestation if pregnancy resulted.

### 2.3. Study Design and Inclusion

**Figure 1:**
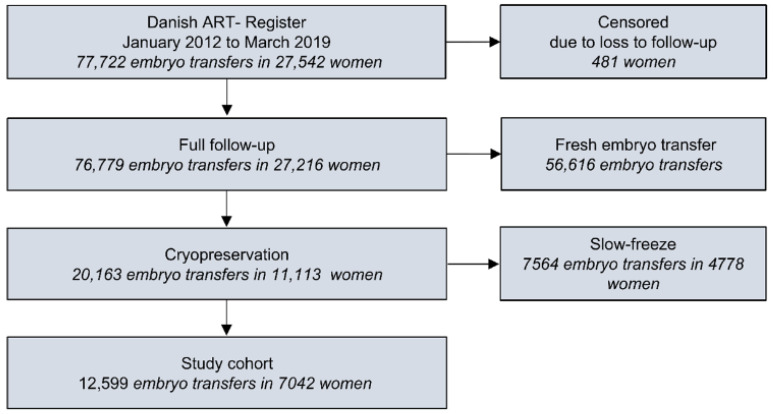
Flowchart of the inclusion of women and embryo transfers from the Danish ART register. ART treatments in this context refer to in vitro fertilization (IVF), with or without intracytoplasmic sperm injection (ICSI), and FET.

The Danish ART register holds data on all ART procedures in public and private clinics and hospitals in Denmark. The study includes all Danish women in the ART register who had undergone at least one embryo transfer and had blastocysts frozen by vitrification (Figure 1).

**Figure 2:**
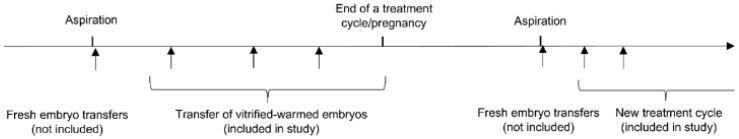
The study setup included embryo transfers after aspiration.

Women may undergo more than one embryo transfer per aspiration. Therefore, we used embryo transfer as the analytical unit [33], stratified by storage time until transfer. If a woman had a new aspiration or gave birth to a child, she could enter the study cohort again (Figure 2). Some women had many transfers followed by the same aspiration. We considered more than 6 transfers as outliers and included only the first 6 transfers that followed an aspiration (87 transfers in 50 women were excluded).

### 2.4. Exposure and Clinical Outcomes

We defined the blastocyst storage time as the exposure defined by the time from the date of aspiration to the date of embryo transfer. The standard procedure for all Danish clinics, during the study period, was vitrification of good-quality blastocysts after 5–6 days of culture; thus the exposure defined as oocyte retrieval to transfer is equivalent to, and a valid proxy for, the period of vitrification to warming.

We grouped the embryos into five groups based on blastocyst storage time: group (i) stored for ≤3 months, group (ii) stored for 4–6 months, group (iii) stored for 7–12 months, group (iv) stored for 13–24 months, and group (v) stored for ≥25 months. We examined the outcomes: biochemical pregnancy, clinical pregnancy, live birth, preterm birth, SGA, LGA, and congenital malformations. Firstly, we assessed biochemical pregnancy based on a positive human chorionic gonadotropin (hCG) test, blood or urine, measured at day 14–16 after embryo transfer. In addition, we analysed the outcomes of clinical pregnancy verified using ultrasound examination approximately 7–8 weeks after the embryo transfer and ultimately live births 140–308 days after the date of embryo transfer [33,34]. Finally, the rates of preterm birth, SGA, LGA, and congenital malformations were analysed for all live births.

### 2.5. Statistical Analyses and Possible Confounders

We examined the influence of blastocyst storage time as a categorical variable, using the shortest storage time of less than or equal to three months (group (i)) as the reference group. Contingency tables on embryo transfers were constructed for the variables according to the five stratification groups.

We used multilevel mixed-effects logistic regression analyses to compute the crude and adjusted relative risk estimates as odds ratios (ORs) with 95% confidence intervals (CIs). The model accounted for multiple embryo transfers in the same woman and oocyte retrieval, and the number of embryo transfers was used to define the longitudinal panel data structure without any other time indication [35,36]. The predictive probability of the three main outcomes, biochemical and clinical pregnancy and live births, was visualized in plots.

Data on potential confounders were obtained from the ART register, besides data on parity and comorbidity, which were obtained from the DMBR and the DNPR. Covariates in the regression models were selected using the modified disjunctive cause criterion [37], and the following adjustment variables were included in the model: the woman’s age at the time of oocyte aspiration in four categories (≤29 years, 30–34 years, 35–39 years, and ≥40 years), calendar year of embryo transfer, body mass index (BMI) in five categories corresponding to the WHO classification (<18.5, 18.5–25, 25–30, 30–35, and ≥35), parity (0, 1 and >2), and smoking (no, yes), alcohol consumption (no, yes (1–2 units), and yes (>2 units)), and comorbidity based on diagnoses from the DNPR according to ICD-codes, including polycystic ovary syndrome, PCOS (ICD-10: E282) and endometriosis (ICD-10: N800-N804). Data were analyzed using Stata/SE 18.0 (StataCorp LLC, College Station, TX, USA).

The study followed the Strengthening the Reporting of Observational Studies in Epidemiology (STROBE) reporting guideline [38]. The study was notified by the Danish Data Protection Agency under the current joint notification of the Region of Southern Denmark (jo. no. 24/15865). Approval was obtained on 1 December 2017 and is valid until 21 December 2028.

Danish law does not require ethical review board approval or patient consent for non-interventional register-based studies.

## 3. Results

### 3.1. Descriptive Characteristics of the Cohort

We identified 7042 unique women with 12,599 FETs (Table 1). The cohort comprised 1047 blastocysts transferred after 13–24 months of storage, and 1097 with ≥25 months of storage before transfer. The longest storage time in our cohort was eleven years and eleven months, and the longest storage time followed by a live-born child was nine years, eleven months. The mean storage time in group (v) was three years and three months. The mean maternal age at oocyte retrieval was 32.2 ± 4.9 years, with the lowest maternal age at oocyte retrieval being for the group with the longest blastocyst storage time, 31.1 ± 4.3 years of storage time. The mean maternal age at embryo transfer was 33.8 ± 4.9 years. The clinical characteristics of the women, including endometrial preparation, at the time of embryo transfer according to blastocyst storage time did not vary except for the occurrence of comorbidity with PCOS, which increased from 2.6% in the reference group to 6.7% for those with blastocysts stored for more than 25 months. 

### 3.2. Clinical Results According to Blastocyst Storage Time

**Figure 3:**
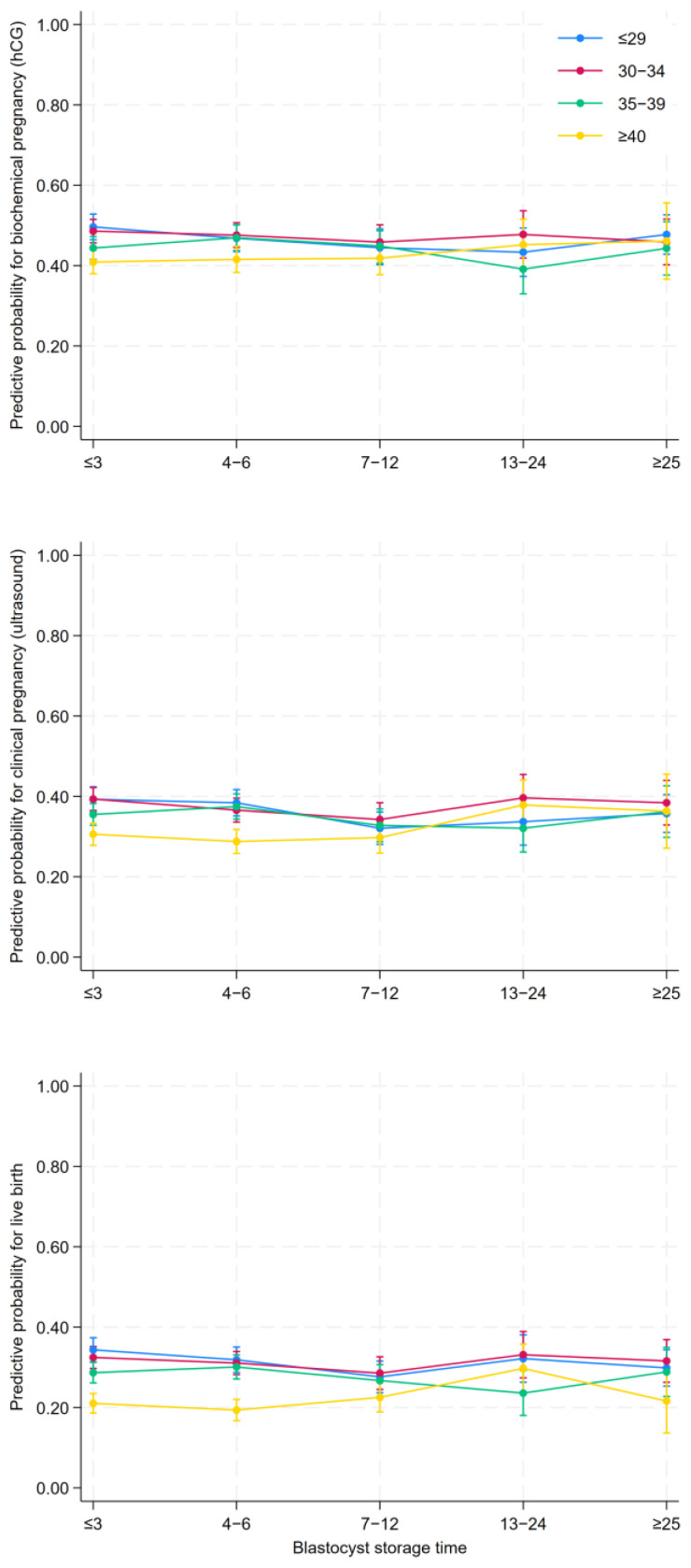
Five categories of storage time and outcomes from the main analysis are presented as visualized predictive probability plots.

Overall, the rate of clinical pregnancy was 35.7%, and the blastocyst storage time did not influence the women’s chance of achieving biochemical pregnancy with aOR from 0.94 (95% CI: 0.80–1.11) to aOR 0.96 (95% CI: 0.82–1.12) in group (iv) and group (v), respectively (Table 2). The chance of a clinical pregnancy did not decrease by storage time aOR 0.96 (95% CI: 0.82–1.13) for storage over >25 months. The chance of a live-born child was 28.6%, and this did not significantly decrease during storage aOR 0.89 (95% CI: 0.75–1.06). Clinical pregnancy and live births showed a slightly decrease in the 7–12-month group but remain unexplained. Sensitivity analyses on alternative storage time groups suggested this decrease was incidental. Additionally, we examined the overall results using blastocyst storage time as a continuous variable rather than a categorical variable, including all embryo transfers after more than four years of storage (*n* = 99 embryo transfers); this did not indicate any decrease over time. The five categories of storage time and outcomes from the main analysis are presented as visualized predictive probability plots in Figure 3.

Furthermore, we assessed birth outcomes based on blastocyst storage time (Table 3). There were no discrepancies in preterm birth, SGA infants, or congenital malformations associated with storage time. However, the risk of LGA was slightly, but non-significantly, increased when comparing the longest storage time with the reference, aOR: 1.42 (95% CI: 0.84–2.42) (Table 3). The most significant confounding variable in all analyses was women’s age at the time of oocyte retrieval. 

## 4. Discussion

### 4.1. Principal Findings

In this extensive nationwide cohort study involving 12.599 frozen embryo transfers (in 7042 women), we found that the association between blastocyst transfer after prolonged vitrification and the chance of biochemical and clinical pregnancies, as well as live births, remained unchanged, despite a slight decrease in clinical pregnancy and live births in the 7–12 month group. Regarding the neonatal outcomes of all live-born children, we found a non-significant trend of increased risk of LGA infants.

In Denmark, fertilized human embryos were previously stored for a maximum of 5 years. However, a recent declaration from the beginning of 2021 changed the storage period until “the time point, of which the woman who will give birth to the child, is no longer eligible to receive assisted reproduction” [39,40]. In Denmark, this means that all blastocysts are eligible for storage until the woman turns 46 years of age. The average woman receiving ART treatment in a public fertility clinic in Denmark is approximately 33 years old; thus, storage time will accordingly increase on average by 8 years to a total of 13 years of storage (if the blastocysts are not utilized). With the introduction of the new declaration, the viability of long-term cryopreserved blastocysts needed to be investigated in a nationwide Danish cohort. 

In our study, patients who underwent their first FET cycle after the fresh blastocyst transfer were included because some patients returned to FET treatment in the hope of having a second FET-conceived child. Thus, the cryo-storage duration of vitrified embryos in this study was much longer than that in some of the other studies, making the results more convincing.

Increased storage time will correspondingly increase maternal age, varying from 18 to 46 years in a Danish population. We adjusted for the women’s age at the time of oocyte aspiration and for the calendar year of embryo transfer.

In this study cohort, there was a slight significant increase in the percentage of embryos from patients with PCOS being stored for a longer period, reflecting more women with PCOS accumulating their embryos, due to high anti-Müllerian hormone, retrieval of more oocytes, risk of ovarian hyperstimulation syndrome, and thus more frequent use of the freeze-all strategy. This observation was also accounted for in the statistical model. We chose to retain odds ratios as the primary effect measures because logistic regression was the prespecified and most appropriate model for our data structure, and sensitivity analysis in the initial study phase using alternative approaches did not materially change the conclusions.

### 4.2. Comparison with Previous Research

Our findings support the observations of the early studies from Wirleitner and Ueno [7,8], as well as the later and larger studies by Cui et al. (2021), Lin et al. (2021), Li et al. (2023), and Ma et al. (2023) [9,10,12,13]. These publications, with FET cycles of vitrified–warmed blastocysts, were divided into timeline groups similar to our cohort and additionally, with the longest storage being 25–98 months, state no differences in pregnancy outcomes nor miscarriage rates from day 5/6 for good-grade blastocysts after cryopreservation [7,8,9,10,12,13]. Like these studies, we did not find any indication of decreased chances of biochemical or clinical pregnancies or live births when comparing longer storage time in stratified categories, with a reference of ≤3 month of vitrification; although clinical pregnancy and live birth rates were slightly lower in the 7–12 month group, this difference lacked statistical precision. Moreover, we found no differences in preterm birth, SGA infants, or congenital malformations associated with storage time. 

The study by Cui et al. (2021) pointed out that postponement of transfer increased the risk of LGA [9]. In secondary analysis, we examined the impact of prolonged storage time on neonatal outcomes, where only the risk of LGA infants was increased. However, the incidence of LGA in the ≥25-month group was very variable due to a small number of LGA cases, and after adjustment for confounders, this increase was non-significant. A larger dataset is needed to draw a firm conclusion. It is well known that infants born after cryopreservation are on average 100 g larger than those conceived after fresh embryo transfer, and the findings, even though non-significant in our study, are not unexpected. The potential cause of the increase in LGA has previously been related to PCOS [41] and epigenetic modifications [42,43,44]. Genetic analysis in vitrified–warmed embryos found several differentially expressed mRNAs and lncRNAs compared with those in fresh embryos. However, the transcriptome between embryos cryopreserved for 3 and 8 years was not differentially expressed, indicating that long-term storage after vitrification does not affect the efficacy and safety of human embryos [45]. Nevertheless, the procedure of vitrification and warming could lead to minor alterations in the transcriptome. However, the proportion of adverse neonatal outcomes did not change significantly with the length of storage duration in these previous studies. 

On the contrary, a negative correlation between storage time and live birth rate in humans has been described [18,19,20,21]. One of the largest studies, a multicentre study by Zhang et al. (2021) including 17.826 women who underwent their first FET following a freeze-all strategy, suggested a decrease in live birth rates after cryopreservation >1 year [19]. Zheng et al. concluded that prolonged freezing did not affect the pregnancy outcomes after transfer of cleavage-stage embryos, whereas storage time of good-quality blastocysts was negatively correlated with FET outcomes in the group with blastocysts transferred [19]. However, only 1245 blastocysts were transferred as single-embryo transfer (SET). Most transfers in this study were performed with double cleavage-state embryos (10,874 embryos) or SET of low-quality embryos (154 cleavage-stage and 415 blastocysts), introducing a significant bias when comparing with studies reporting on only good-quality SET [19].

Several other studies add to this concern. The study from Hu et al. (2022) divided 15.000 FET cycles into a few months’ differences [20]. Also, in this study, most of the vitrified embryos were cleavage-stage embryos. However, Hu et al. (2022) [20] did not distinguish between cleavage-stage embryos and blastocysts in their result section. They reported a higher LBR at 3–4 months of storage, and prolonged storage time of >6 months was associated with reduced pregnancy rates [20]. Three studies, with storage groups similar to our classification, concluded that the embryo survival rate decreased significantly with longer durations of cryopreservation [18,21,22]. However, no results were presented on LBRs in the study from Mao et al. (2022) [21]. The studies from Li et al. (2020) and Zheng et al. (2023) described a significantly decreased clinical pregnancy rate and live birth rate with increasing storage time [18,22]. However, the study from Li et al. (2020) includes both vitrified blastocysts and cleavage-stage embryos and does not distinguish between these two types of human embryos in the results, which could introduce a bias when comparing with studies reporting on only vitrified blastocysts [18]. Another study from Cimadomo et al. (2022) reported a lower live birth rate (LBR) in the groups with prolonged storage; however, this study included all FET cycles; thus, a reasonable explanation is due to top-quality embryos being transferred first, when more blastocysts were available, thereby leaving the lower-quality blastocysts for subsequent thaw and transfers [11].

The mechanisms that could describe a negative correlation are unknown. Some studies indicate that freezing can affect the embryonic cytoskeleton and alter the transcriptome of embryos and subsequently contribute to impaired implantation potential of frozen–thawed embryos [46,47].

According to all these studies, patients who adopt a freeze-all strategy should be advised that early FET might achieve a better outcome in terms of pregnancy rates and live births. However, importantly, similarly to all these studies, no effect on miscarriage was observed [18,19,20,22].

Despite conflicting results in the literature, the topic is of great importance due to the more advanced technologies and improved legislation advocating for longer vitrification times.

### 4.3. Strengths and Limitations

Strengths of this study include the large sample size, the nationwide cohort study design with complete data, the adjustment of multiple covariates such as calendar year of treatment and age of the woman at the time of oocyte retrieval, and the uniform organization of the healthcare system, including data from all public and private fertility clinics across the entire country from more than a decade. Furthermore, we could classify the storage time into relevant cryoprotection time groups, as both continuous and categorical variables, and with valid outcome measurements, i.e., biochemical, clinical pregnancy, and live births. Further studies are warranted to verify our null finding of no association between long storage time and pregnancy and birth outcomes in other populations with more embryos with the most prolonged storage time.

### 4.4. Implications

Determining whether the technologies offering prolonged storage time of the embryos have an impact on implantation, pregnancy, and live births has important implications for the organization, and given our null findings, the implementation of the new legislation of prolonged cryopreservation time and the offer of free ART treatment up to the second child in Denmark are reassuring. However, the slightly increased risk of LGA neonates after longer cryopreservation should be given attention, as the condition is associated with an increased risk of serious adverse birth outcomes. Also, the study needs to be replicated when the data is more robust in the latter storage group.

## 5. Conclusions

In conclusion, our findings suggest that a long storage time of embryos was not associated with decreased chances of achieving a pregnancy, biochemical or clinical, or a live birth.

## Figures and Tables

**Table 1:** Descriptive characteristics of embryo transfers using vitrification in women undergoing assisted reproductive technology (ART) with blastocysts from 1 January 2012 through 1 March 2019.

	Blastocyst Storage Time									
	≤3 Months		4–6 Months		7–12 Months		13–24 Months		≥25 Months	
Embryo transfers (women)	4430	(3716)	3820	(3036)	2205	(1642)	1047	(793)	1097	(763)
Maternal age at oocyte retrieval, mean (SD)	33.6	(4.8)	33.3	(4.8)	33.2	(5.0)	32.9	(4.9)	31.1	(4.3)
≤29	976	(22.0%)	898	(23.5%)	555	(25.2%)	270	(25.8%)	442	(40.3%)
30–34	1131	(25.5%)	1049	(27.5%)	534	(24.2%)	285	(27.2%)	318	(29.0%)
35–39	1199	(27.1%)	959	(25.1%)	541	(24.5%)	251	(24.0%)	227	(20.7%)
≥40	1124	(25.4%)	914	(23.9%)	575	(26.1%)	241	(23.0%)	110	(10.0%)
Material age at embryo transfer	33.8	(4.9)	33.6	(4.9)	33.9	(5.0)	34.3	(4.9)	34.4	(4.4)
Paternal age, mean (SD)	41.8	(19.5)	41.0	(18.5)	40.7	(17.3)	40.5	(16.2)	39.8	(14.4)
Course of infertility, *n* (%)										
Female factor	533	(12.0%)	400	(10.5%)	203	(9.2%)	81	(7.7%)	71	(6.5%)
Male factor	1424	(32.1%)	1231	(32.2%)	676	(30.7%)	307	(29.3%)	405	(36.9%)
Female/male and idiopathic	2473	(55.8%)	2189	(57.3%)	1326	(60.1%)	659	(62.9%)	621	(56.6%)
Fertilization method, *n* (%)										
IVF	2232	(50.4%)	1886	(49.4%)	1094	(49.6%)	464	(44.3%)	513	(46.8%)
ICSI	2060	(46.5%)	1788	(46.8%)	1040	(47.2%)	557	(53.2%)	560	(51.0%)
IVF + ICSI	20	(0.5%)	25	(0.7%)	15	(0.7%)	<5	(-)	8	(0.7%)
Testicular sperm aspiration	118	(2.7%)	121	(3.2%)	56	(2.5%)	21	(2.0%)	16	(1.5%)
Embryos transferred, *n* (%)										
1	3941	(89.0%)	3358	(87.9%)	1964	(89.1%)	917	(87.6%)	965	(88.0%)
≥2	489	(11.0%)	462	(12.1%)	241	(10.9%)	130	(12.4%)	132	(12.0%)
Parity										
0	3005	(67.8%)	2778	(72.7%)	1694	(76.8%)	722	(69.0%)	218	(19.9%)
1	1227	(27.7%)	892	(23.4%)	421	(19.1%)	287	(27.4%)	755	(68.8%)
>2	198	(4.5%)	150	(3.9%)	90	(4.1%)	38	(3.6%)	124	(11.3%)
Previous embryos transferred, *n* (%)										
0	4191	(94.6%)	2479	(64.9%)	849	(38.5%)	403	(38.5%)	692	(63.1%)
1	239	(5.4%)	1312	(34.3%)	1194	(54.1%)	460	(43.9%)	329	(30.0%)
≥2	0	(0.0%)	29	(0.8%)	162	(7.3%)	184	(17.6%)	76	(6.9%)
Hormone treatment										
hCG Natural cycles	954	(21.5%)	763	(20.0%)	364	(16.5%)	146	(13.9%)	123	(11.2%)
Progesterone/estrogen Substituted cycles	1348	(30.4%)	1138	(29.8%)	630	(28.6%)	318	(30.4%)	341	(31.1%)
Other Stimulatated cycle	2100	(47.4%)	1899	(49.7%)	1193	(54.1%)	567	(54.2%)	625	(57.0%)
Missing	28	(0.6%)	20	(0.5%)	18	(0.8%)	16	(1.5%)	8	(0.7%)
BMI, N (%)										
<18.5 (underweight)	139	(3.1%)	136	(3.6%)	58	(2.6%)	24	(2.3%)	38	(3.5%)
18.5–25 (normal)	2385	(53.8%)	2036	(53.3%)	1169	(53.0%)	513	(49.0%)	529	(48.2%)
25–30 (pre-obesity)	903	(20.4%)	757	(19.8%)	432	(19.6%)	258	(24.6%)	216	(19.7%)
30–35 (obese I)	280	(6.3%)	275	(7.2%)	151	(6.8%)	73	(7.0%)	89	(8.1%)
≥35 (obese II-III)	51	(1.2%)	61	(1.6%)	25	(1.1%)	19	(1.8%)	18	(1.6%)
BMI missing	672	(15.2%)	555	(14.5%)	370	(16.8%)	160	(15.3%)	207	(18.9%)
Smoking at the time of embryo transfer, N (%)										
No	3803	(85.8%)	3232	(84.6%)	1812	(82.2%)	854	(81.6%)	944	(86.1%)
Yes	258	(5.8%)	244	(6.4%)	141	(6.4%)	69	(6.6%)	68	(6.2%)
Missing	369	(8.3%)	344	(9.0%)	252	(11.4%)	124	(11.8%)	85	(7.7%)
Alcohol at the time of embryo transfer, N (%)										
No	2002	(45.2%)	1735	(45.4%)	1002	(45.4%)	499	(47.7%)	494	(45.0%)
Yes (1–2 units)	991	(22.4%)	880	(23.0%)	480	(21.8%)	213	(20.3%)	246	(22.4%)
Yes (>2 units)	678	(15.3%)	570	(14.9%)	317	(14.4%)	154	(14.7%)	156	(14.2%)
Missing	759	(17.1%)	635	(16.6%)	406	(18.4%)	181	(17.3%)	201	(18.3%)
Comorbidity										
Polycystic ovary syndrome, PCOS	116	(2.6%)	131	(3.4%)	96	(4.4%)	54	(5.2%)	73	(6.7%)
Endometrioses	274	(6.2%)	212	(5.5%)	149	(6.8%)	92	(8.8%)	78	(7.1%)
The calendar year of infertility treatment, N (%)										
2012–2013	439	(9.9%)	307	(8.0%)	121	(5.5%)	67	(6.4%)	91	(8.3%)
2014–2015	1012	(22.8%)	851	(22.3%)	441	(20.0%)	162	(15.5%)	194	(17.7%)
2016–2017	1770	(40.0%)	1558	(40.8%)	906	(41.1%)	401	(38.3%)	404	(36.8%)
2018–2019	1209	(27.3%)	1104	(28.9%)	737	(33.4%)	417	(39.8%)	408	(37.2%)

**Table 2:** Odds ratios for biochemical pregnancy, clinical pregnancy, and live births according to blastocyst storage time in embryo transfers from 1 January 2012 to 31 March 2019.

	Blastocyst Storage Time				
	≤3 Months	4–6 Months	7–12 Months	13–24 Months	≥25 Months
Biochemical pregnancy (hCG)	2.007 (45.3%)	1.701 (44.5%)	939 (42.4%)	444 (42.4%)	502 (45.8%)
OR	Ref.	1.00 (0.91–1.09)	0.93 (0.83–1.03)	0.91 (0.78–1.05)	1.02 (0.88–1.18)
aOR ^a^	Ref.	1.01 (0.90–1.09)	0.95 (0.84–1.08)	0.94 (0.80–1.11)	0.96 (0.82–1.12)
Clinical Pregnancy (ultrasound)	1.583 (35.7%)	1302 (34.1%)	667 (30.2%)	350 (33.4%)	397 (36.2%)
OR	Ref.	0.96 (0.87–1.05)	0.81 (0.72–0.91)	0.95 (0.82–1.11)	1.03 (0.89–1.20)
aOR ^a^	Ref.	0.99 (0.89–1.09)	0.86 (0.76–0.98)	1.01 (0.86–1.19)	0.96 (0.82–1.13)
Live birth	1.267 (28.6%)	1.022 (26.8%)	524 (23.8%)	276 (26.4%)	312 (28.3%)
OR	Ref.	0.94 (0.85–1.05)	0.84 (0.74–0.96)	1.00 (0.84–1.19)	1.02 (0.87–1.20)
aOR ^a^	Ref.	0.97 (0.87–1.08)	0.88 (0.76–1.01)	1.03 (0.86–1.23)	0.89 (0.75–1.06)

^a^: Adjusted for the women’s age at the time of oocyte aspiration, calendar year of treatment, BMI, parity, smoking, alcohol, polycystic ovary syndrome (PCOS), and endometrioses.

**Table 3:** Odds ratios for preterm birth, small and large for gestational age, and congenital malformations in live-born singletons according to blastocyst storage time in embryo transfers from 1 January 2012 to 31 May 2018 *.

	Blastocyst Storage Time				
	≤3 Months	4–6 Months	7–12 Months	13–24 Months	≥25 Months
Preterm birth	78 (8.0%)	66 (8.4%)	32 (8.3%)	11 (6.0%)	10 (4.5%)
OR	Ref.	1.06 (0.76–1.50)	1.01 (0.71–1.68)	0.74 (0.39–1.43)	0.55 (0.27–1.06)
aOR ^a^	Ref.	0.98 (0.69–1.40)	1.07 (0.69–1.64)	0.75 (0.39–1.46)	0.56 (0.28–1.15)
Small for gestational age ^b^	149 (15.2%)	113 (14.4%)	53 (14.3)	25 (13.7%)	23 (10.4%)
OR	Ref.	0.94 (0.72–1.22)	0.94 (0.67–1.32)	0.89 (0.56–1.40)	0.65 (0.41–1.06)
aOR ^a^	Ref.	0.92 (0.71–1.21)	0.92 (0.65–1.30)	0.93 (0.58–1.49)	1.02 (0.61–1.68)
Large for gestational age ^b^	59 (6.0%)	59 (7.5%)	21 (5.7%)	18 (9.9%)	26 (11.7%)
OR	Ref.	1.27 (0.87–1.84)	0.94 (0.57–1.58)	1.71 (0.98–2.97)	2.11 (1.29–3.42)
aOR ^a^	Ref.	1.24 (0.84–1.81)	0.93 (0.55–1.57)	1.54 (0.88–2.72)	1.42 (0.84–2.42)
Congenital malformations	98 (1.00%)	75 (9.6%)	35 (9.4%)	20 (10.1%)	18 (8.1%)
OR	Ref.	0.95 (0.69–1.31)	0.93 (0.63–1.41)	1.11 (0.67–1.85)	0.80 (0.47–1.34)
aOR ^a^	Ref.	0.95 (0.69–1.30)	0.88 (0.58–1.33)	1.15 (0.68–1.92)	1.00 (0.57–1.75)

* Study period restricted due to lack of an updated medical birth register. ^a^: Adjusted for the women’s age at the time of oocyte aspiration, calendar year of treatment, BMI, parity, smoking, alcohol, polycystic ovary syndrome (PCOS), and endometrioses. ^b^: Missing: SGA: 23.1%, LGA 23.1%.

## Data Availability

Given Danish legislation, our approval to use these data for the current study does not allow us to distribute or make patient data directly available to other parties. The authors of this paper have no special access privileges to the data used in this study, and other researchers may apply for access to the data through an application to the Research Service at the Danish Health Data Authority.

## Abbreviations

The following abbreviations are used in this manuscript:

ART	Assisted Reproductive Technology
FET	Frozen Embryo Transfers
LGA	Large for Gestational Age
SGA	Small for Gestational Age
OHSS	Ovarian Hyperstimulation Syndrome
PCOS	Polycystic Ovarian Syndrome
hCG	human Chorionic Gonadotropin
LN_2_	Liquid Nitrogen
DNPR	Danish National Patient Register
DMBR	Danish Medical Birth Register

## Appendix A. ICD-10 Diagnoses of Congenital Malformation

Q10–Q16
Q20–Q28
Q30–Q34
Q35–Q37
Q38–Q45
Q50–Q56
Q60–Q64
Q65–Q79
Q80–Q89
Q90–Q99

## References

[1] Trounson A., Mohr L. (1983). Human pregnancy following cryopreservation, thawing and transfer of an eight-cell embryo. Nature.

[2] Rodriguez-Wallberg K.A., Waterstone M., Anastacio A. (2019). Ice age: Cryopreservation in assisted reproduction—An update. Reprod. Biol..

[3] Stormlund S., Sopa N., Zedeler A., Bogstad J., Praetorius L., Nielsen H.S., Kitlinski M.L., Skouby S.O., Mikkelsen A.L., Spangmose A.L. (2020). Freeze-all versus fresh blastocyst transfer strategy during in vitro fertilisation in women with regular menstrual cycles: Multicentre randomised controlled trial. BMJ.

[4] Devroey P., Polyzos N.P., Blockeel C. (2011). An OHSS-Free Clinic by segmentation of IVF treatment. Hum. Reprod..

[5] Wyns C., De Geyter C., Calhaz-Jorge C., Kupka M.S., Motrenko T., Smeenk J., Bergh C., Tandler-Schneider A., Rugescu I.A., European IVF Monitoring Consortium (EIM), for the European Society of Human Reproduction and Embryology (ESHRE) (2022). ART in Europe, 2018: Results generated from European registries by ESHRE. Hum. Reprod. Open.

[6] Rienzi L., Gracia C., Maggiulli R., LaBarbera A.R., Kaser D.J., Ubaldi F.M., Vanderpoel S., Racowsky C. (2017). Oocyte, embryo and blastocyst cryopreservation in ART: Systematic review and meta-analysis comparing slow-freezing versus vitrification to produce evidence for the development of global guidance. Hum. Reprod. Update.

[7] Wirleitner B., Vanderzwalmen P., Bach M., Baramsai B., Neyer A., Schwerda D., Schuff M., Spitzer D., Stecher A., Zintz M. (2013). The time aspect in storing vitrified blastocysts: Its impact on survival rate, implantation potential and babies born. Hum. Reprod..

[8] Ueno S., Uchiyama K., Kuroda T., Yabuuchi A., Ezoe K., Okimura T., Okuno T., Kobayashi T., Kato K. (2018). Cryostorage duration does not affect pregnancy and neonatal outcomes: A retrospective single-centre cohort study of vitrified-warmed blastocysts. Reprod. Biomed. Online.

[9] Cui M., Dong X., Lyu S., Zheng Y., Ai J. (2021). The Impact of Embryo Storage Time on Pregnancy and Perinatal Outcomes and the Time Limit of Vitrification: A Retrospective Cohort Study. Front. Endocrinol..

[10] Lin R., Zhou H., Wang C., Chen H., Shu J., Gan X., Xu K., Zhao X. (2021). Does longer storage of blastocysts with equal grades in a cryopreserved state affect the perinatal outcomes?. Cryobiology.

[11] Cimadomo D., Fabozzi G., Dovere L., Maggiulli R., Albricci L., Innocenti F., Soscia D., Giancani A., Vaiarelli A., Guido M. (2022). Clinical, obstetric and perinatal outcomes after vitrified-warmed euploid blastocyst transfer are independent of cryo-storage duration. Reprod. Biomed. Online.

[12] Li X., Guo P., Blockeel C., Li X., Deng L., Yang J., Li C., Lin M., Wu H., Cai G. (2023). Storage duration of vitrified embryos does not affect pregnancy and neonatal outcomes after frozen-thawed embryo transfer. Front. Endocrinol..

[13] Ma Y., Sun M., Wen T., Ding C., Liu L.W., Meng T., Song J., Hou X., Mai Q., Xu Y. (2023). Storage time does not influence pregnancy and neonatal outcomes for first single vitrified high-quality blastocyst transfer cycle. Reprod. Biomed. Online.

[14] Youssry M., Ozmen B., Zohni K., Diedrich K., Al-Hasani S. (2008). Current aspects of blastocyst cryopreservation. Reprod. Biomed. Online.

[15] Lattes K., Checa M.A., Vassena R., Brassesco M., Vernaeve V. (2017). There is no evidence that the time from egg retrieval to embryo transfer affects live birth rates in a freeze-all strategy. Hum. Reprod..

[16] Malter H. (2018). Life Interrupted: The Nature and Consequences of Cryostasis. Semin. Reprod. Med..

[17] Canosa S., Cimadomo D., Conforti A., Maggiulli R., Giancani A., Tallarita A., Golia F., Fabozzi G., Vaiarelli A., Gennarelli G. (2022). The effect of extended cryo-storage following vitrification on embryo competence: A systematic review and meta-analysis. J. Assist. Reprod. Genet..

[18] Li J., Yin M., Wang B., Lin J., Chen Q., Wang N., Lyu Q., Wang Y., Kuang Y., Zhu Q. (2020). The effect of storage time after vitrification on pregnancy and neonatal outcomes among 24 698 patients following the first embryo transfer cycles. Hum. Reprod..

[19] Zhang X., Wu S., Hao G., Wu X., Ren H., Zhang Y., Yang A., Bi X., Bai L., Zhang Y. (2021). Prolonged Cryopreservation Negatively Affects Embryo Transfer Outcomes Following the Elective Freeze-All Strategy: A Multicenter Retrospective Study. Front. Endocrinol..

[20] Hu K.L., Hunt S., Zhang D., Li R., Mol B.W. (2022). The association between embryo storage time and treatment success in women undergoing freeze-all embryo transfer. Fertil. Steril..

[21] Mao Y., Tang N., Luo Y., Yin P., Li L. (2022). Effects of vitrified cryopreservation duration on IVF and neonatal outcomes. J. Ovarian Res..

[22] Zheng Q., Mo M., Zhang H., Xu S., Xu F., Wang S., Zeng Y. (2023). Prolong cryopreservation duration negatively affects pregnancy outcomes of vitrified-warmed blastocyst transfers using an open-device system: A retrospective cohort study. Eur. J. Obstet. Gynecol. Reprod. Biol..

[23] Zhan S., Lin C., Lin Q., Gan J., Wang C., Luo Y., Liu J., Du H., Liu H. (2024). Vitrification preservation of good-quality blastocysts for more than 5 years reduces implantation and live birth rates. Hum. Reprod..

[24] Schmidt M., Schmidt S.A., Sandegaard J.L., Ehrenstein V., Pedersen L., Sorensen H.T. (2015). The Danish National Patient Registry: A review of content, data quality, and research potential. Clin. Epidemiol..

[25] Andersen A.N., Westergaard H.B., Olsen J. (1999). The Danish in vitro fertilisation (IVF) register. Dan. Med. Bull..

[26] Westergaard H.B., Johansen A.M., Erb K., Andersen A.N. (2000). Danish National IVF Registry 1994 and 1995. Treatment, pregnancy outcome and complications during pregnancy. Acta Obstet. Gynecol. Scand..

[27] Jolving L.R., Erb K., Norgard B.M., Fedder J., Larsen M.D. (2021). The Danish National Register of assisted reproductive technology: Content and research potentials. Eur. J. Epidemiol..

[28] Kristensen J., Langhoff-Roos J., Skovgaard L.T., Kristensen F.B. (1996). Validation of the Danish Birth Registration. J. Clin. Epidemiol..

[29] Bliddal M., Broe A., Pottegard A., Olsen J., Langhoff-Roos J. (2018). The Danish Medical Birth Register. Eur. J. Epidemiol..

[30] (2011). Alpha Scientists in Reproductive Medicine; ESHRE Special Interest Group of Embryology. The Istanbul consensus workshop on embryo assessment: Proceedings of an expert meeting. Hum. Reprod..

[31] Coticchio G., Ahlstrom A., Arroyo G., Balaban B., Campbell A., De Los Santos M.J., Ebner T., Gardner D.K., Kovacic B., Working Group on the Update of the ESHRE/ALPHA Istanbul Consensus (2025). The Istanbul consensus update: A revised ESHRE/ALPHA consensus on oocyte and embryo static and dynamic morphological assessmentdagger, double dagger. Hum. Reprod..

[32] Du T., Chen H., Fu R., Chen Q., Wang Y., Mol B.W., Kuang Y., Lyu Q. (2017). Comparison of ectopic pregnancy risk among transfers of embryos vitrified on day 3, day 5, and day 6. Fertil. Steril..

[33] Norgard B.M., Catalini L., Jolving L.R., Larsen M.D., Friedman S., Fedder J. (2021). The Efficacy of Assisted Reproduction in Women with a Wide Spectrum of Chronic Diseases—A Review. Clin. Epidemiol..

[34] Friedman S., Larsen P.V., Fedder J., Norgard B.M. (2017). The reduced chance of a live birth in women with IBD receiving assisted reproduction is due to a failure to achieve a clinical pregnancy. Gut.

[35] Messerlian C., Gaskins A.J. (2017). Epidemiologic Approaches for Studying Assisted Reproductive Technologies: Design, Methods, Analysis and Interpretation. Curr. Epidemiol. Rep..

[36] Schober P., Vetter T.R. (2018). Repeated Measures Designs and Analysis of Longitudinal Data: If at First You Do Not Succeed-Try, Try Again. Anesth. Analg..

[37] VanderWeele T.J. (2019). Principles of confounder selection. Eur. J. Epidemiol..

[38] von Elm E., Altman D.G., Egger M., Pocock S.J., Gotzsche P.C., Vandenbroucke J.P., Initiative S. (2008). The Strengthening the Reporting of Observational Studies in Epidemiology (STROBE) statement: Guidelines for reporting observational studies. J. Clin. Epidemiol..

[39] Sundhedsministeriet (2021). Lov om Ændring af lov om Assisteret Reproduktion i Forbindelse Med Behandling, Diagnostik og Forskning m.v. LOV nr 129 af 30/01/2021. https://www.retsinformation.dk/eli/lta/2021/129.

[40] Sundhedsministeriet (2019). Bekendtgørelse af lov om Assisteret Reproduktion i Forbindelse Med Behandling, Diagnostik og Forskning m.v. LBK nr 902 af 23/08/2019. https://www.retsinformation.dk/eli/lta/2019/902.

[41] Chen X., Gissler M., Lavebratt C. (2023). Birth outcomes in mothers with hypertensive disorders and polycystic ovary syndrome: A population-based cohort study. Hum. Reprod. Open.

[42] Liu S.Y., Teng B., Fu J., Li X., Zheng Y., Sun X.X. (2013). Obstetric and neonatal outcomes after transfer of vitrified early cleavage embryos. Hum. Reprod..

[43] Litzky J.F., Boulet S.L., Esfandiari N., Zhang Y., Kissin D.M., Theiler R.N., Marsit C.J. (2018). Effect of frozen/thawed embryo transfer on birthweight, macrosomia, and low birthweight rates in US singleton infants. Am. J. Obstet. Gynecol..

[44] Ginstrom Ernstad E., Wennerholm U.B., Khatibi A., Petzold M., Bergh C. (2019). Neonatal and maternal outcome after frozen embryo transfer: Increased risks in programmed cycles. Am. J. Obstet. Gynecol..

[45] Li J., Zhu L., Huang J., Liu W., Han W., Huang G. (2021). Long-Term Storage Does Not Affect the Expression Profiles of mRNA and Long Non-Coding RNA in Vitrified-Warmed Human Embryos. Front. Genet..

[46] Tharasanit T., Colenbrander B., Stout T.A. (2005). Effect of cryopreservation on the cellular integrity of equine embryos. Reproduction.

[47] Kopeika J., Thornhill A., Khalaf Y. (2015). The effect of cryopreservation on the genome of gametes and embryos: Principles of cryobiology and critical appraisal of the evidence. Hum. Reprod. Update.

